# Mutualistic Interactions Drive Ecological Niche Convergence in a Diverse Butterfly Community

**DOI:** 10.1371/journal.pbio.0060300

**Published:** 2008-12-02

**Authors:** Marianne Elias, Zachariah Gompert, Chris Jiggins, Keith Willmott

**Affiliations:** 1 Institute of Evolutionary Biology, University of Edinburgh, Edinburgh, United Kingdom; 2 Department of Zoology, University of Cambridge, Cambridge, United Kingdom; 3 Department of Botany, University of Wyoming, Laramie, Wyoming, United States of America; 4 McGuire Center for Lepidoptera and Biodiversity, Florida Museum of Natural History, University of Florida, Gainesville, Florida, United States of America; Cornell University, United States of America

## Abstract

Ecological communities are structured in part by evolutionary interactions among their members. A number of recent studies incorporating phylogenetics into community ecology have upheld the paradigm that competition drives ecological divergence among species of the same guild. However, the role of other interspecific interactions, in particular positive interactions such as mutualism, remains poorly explored. We characterized the ecological niche and inferred phylogenetic relationships among members of a diverse community of neotropical Müllerian mimetic butterflies. Müllerian mimicry is one of the best studied examples of mutualism, in which unpalatable species converge in wing pattern locally to advertize their toxicity to predators. We provide evidence that mutualistic interactions can drive convergence along multiple ecological axes, outweighing both phylogeny and competition in shaping community structure. Our findings imply that ecological communities are adaptively assembled to a much greater degree than commonly suspected. In addition, our results show that phenotype and ecology are strongly linked and support the idea that mimicry can cause ecological speciation through multiple cascading effects on species' biology.

## Author Summary

What governs the composition of communities of species? Competition promotes divergence in behavior and habitat, allowing species to co-exist. But the effects of other interactions, such as mutualism, are less well understood. We examined the interplay between mutualistic interactions, common ancestry and competition in mimetic butterflies, one of the best studied examples of mutualism, in which species converge in wing pattern to advertize their toxicity to predators. We showed that mutualism drives convergence in flight height and forest habitat, and that these effects outweigh common ancestry (which should lead related species to be more similar) and competition (which promotes ecological divergence). Our findings imply that species that benefit from one another might evolve to form more tightly knit local communities, suggesting that adaptation is a more important process affecting community composition than is commonly suspected. Our results also support the idea that mimicry can cause speciation, through its multiple cascading effects on species' biology.

## Abstract 3

Müllerian mimicry, a classic mutualism, is associated with microhabitat convergence in tropical butterflies, outweighing both common ancestry and competition. Positive interactions may thus be more important in community assembly than commonly assumed.

## Introduction

A recent review of community ecology literature has suggested that “there is a dynamic interplay between ecology and evolution within communities” [1], such that understanding community structure requires study of both the evolutionary history and recent interactions between constituent species [2–4]. In this context, competition is usually seen as the major force shaping community ecology [5,6], causing ecological displacement among interacting species in communities or biasing community assembly towards more ecologically divergent species [7–10]. However, recent work has uncovered the importance of positive interspecific interactions in influencing the composition and ecological structure of communities [11–15], suggesting that such interactions could outweigh competition [16], for example by facilitating colonization by invasive species [17]. Here we explore the hypothesis that mutualistic interactions among species that are ecologically similar in a broad sense (i.e., members of the same guild) can result in convergence along multiple fine-scale ecological variables.

Müllerian mimicry is a spectacular example of ecological adaptation that is mutually beneficial to multiple species [18,19]. Butterflies are perhaps the best studied Müllerian mimetic organisms [20], where unpalatable species have evolved brightly colored wing patterns that advertize their toxicity to predators. Multiple co-occurring species converge in wing pattern, forming mimicry complexes (i.e., sets of species sharing the same wing pattern), and thus share the cost of educating predators [21]. However, although Müllerian mimicry favors convergence in warning signal locally, ten or more distinct mimicry complexes may coexist in rainforest butterfly communities [22,23]. There is evidence that mimicry complexes are partially segregated by microhabitat such that co-mimics (species that belong to the same mimicry complex) tend to occur in similar microhabitats [24–27]. Key predators including insectivorous birds are likely segregated in a similar way [28], which in turn should reduce selection for convergence in wing pattern across microhabitats, facilitating the stable coexistence of several mimicry complexes in communities [18,29]. Mimicry should therefore promote adaptive convergence in microhabitat niche among co-mimic species to maximize warning signal overlap, thereby counteracting competition within mimicry complexes. To date, however, the microhabitat niche of mimetic butterflies has never been studied in a phylogenetic context, and ecological similarity among co-mimics could be largely due to common ancestry.

Here we investigate the evolution of microhabitat use among a diverse community of Müllerian mimetic butterflies and conduct the first test of adaptive ecological convergence at the community level. By examining multiple niche axes simultaneously for butterfly species belonging to the same and different mimicry complexes we are able to disentangle the interplay between mimicry, common ancestry and competition in the evolution of microhabitat niche. Our study group is the ithomiines (Nymphalidae: Ithomiinae), the most diverse (∼350 species [30]) and abundant Müllerian mimetic butterflies in the neotropics, which act as mimicry models for many other Lepidoptera species [24,31]. Ithomiine larvae feed almost exclusively on Solanaceae plants [32], and adult males of nearly all species actively seek sources of pyrrolizidine alkaloids to provide chemical protection from predators [33] as well as sex pheromone precursors [34]. Exploitation of such a relatively narrow range of resources among diverse taxa makes ithomiines an ideal system in which to investigate the relative roles of competition and mutualistic interactions in shaping community structure.

## Results/Discussion

**Figure 1:**
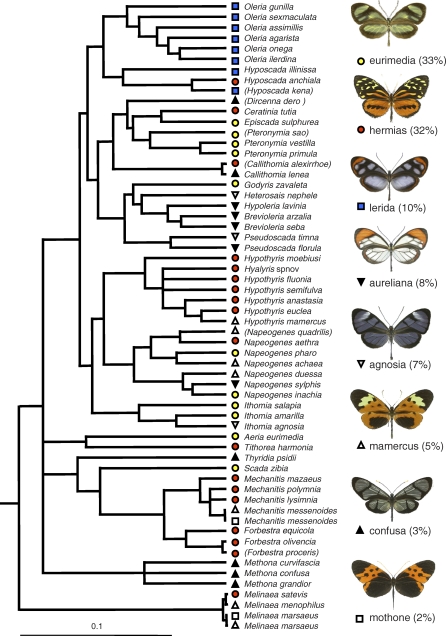
Phylogeny of the Ithomiine Species of the Community The relaxed-clock tree (maximum clade credibility tree resulting from a Bayesian phylogenetic analysis using a mitochondrial region and a nuclear gene) shows the 60 ithomiine taxa (58 species) of the community, after pruning 20 additional taxa not present in the community. All nodes have a posterior probability above 0.90. Brackets indicate rare species that were excluded from the analyses because not all microhabitat variables could be measured. The eight mimetic patterns are shown on the right with their names and relative abundance in the community, and indicated by colored symbols at the tips of the tree (see Table S1 for the list of taxa and corresponding mimetic patterns). There was a significant phylogenetic signal in the mimicry structure of the community (*r* = 0.162, *n* = 1,431, *p* < 0.0001), confirming that closely related species share color patterns more often than expected at random.

The study community in the upper Amazon contained 58 ithomiine species [35] distributed among eight mimicry complexes (Figure 1). A complete species phylogeny for the community was generated from 3,511 bp of mitochondrial and nuclear DNA sequence (Figure 1, Table S1). Microhabitat variables representing forest structure, topography (ridge versus valley) and flight height were recorded during a 3-month field study of the ithomiine community. Forest structure was assessed using 16 variables designed to capture variation in vegetation density, height and light levels, which were subsequently summarized by three principal components, FS1, FS2 and FS3 (Table S2).

We first investigated whether mimicry complexes were segregated by microhabitat. The abundance of individuals in distinct mimicry complexes was significantly segregated along each variable (ANOVAs; FS1: F_7,947_ = 72.277, FS2: F_7,947_ = 7.791, FS3: F_7,947_ = 5.226, Topography: F_7,597_ = 28.318, Flight height: F_7,1215_ = 24.891; *p* ≤ 0.0001 for all variables) and in the multidimensional microhabitat space constituted by the five variables measured (hereafter, global microhabitat; MANOVA: Pillai–Bartlett statistic_35,4735_ = 1.594, *p* < 0.0001). Thus, individuals are more likely to be found alongside others that share the same color pattern than expected by chance. Furthermore, the same is true at a species level, such that segregation is not solely driven by the most abundant species. Indeed, regardless of relative abundance, species were segregated according to mimicry complex along FS1 (ANOVA: F_7,46_ = 5.519, *p* = 0.0013) and flight height (F_7,46_ = 14.648, *p* < 0.0001), and for the global microhabitat (MANOVA: Pillai–Bartlett statistic_35,230_ = 1.594, *p* < 0.0001), meaning that co-mimic species were ecologically more similar than expected at random. Thus, mimicry complexes were segregated in multiple microhabitat dimensions in the study community, in line with previous observations in other communities [24–27].

**Figure 2:**
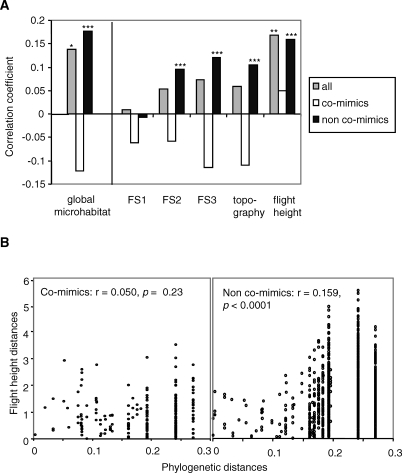
Correlation Between Ecological and Phylogenetic Distances (A) Correlation coefficients among all species (gray, *n* = 1,431), among co-mimics (white, *n* = 225) and among non-co-mimics (black, *n* = 1,206) for the global microhabitat and for each ecological variable. One-tailed *p*-values for positive correlation are shown for all comparisons with a significant correlation: *, *p* < 0.05, **, *p* < 0.01, ***, *p* < 0.001 (tests performed using 10,000 permutations, all significant after correction for multiple tests). (B) Scatterplots showing the relationship between flight-height distances and phylogenetic distances for co-mimics and for non-co-mimics, as an example illustrating the results above. The correlation is significant for non-co-mimics only.

We then investigated the influence of phylogeny on microhabitat use. Both global microhabitat and distances along each individual microhabitat variable correlated positively with phylogenetic distances (Figure 2), but correlations were significantly supported only for flight height (*r =* 0.168, *n* = 1,431, *p* = 0.0032) and global microhabitat (*r =* 0.137, *n* = 1,431, *p* = 0.0262, Figure 2). Thus, as species diverge genetically they also diverge ecologically, as might be expected, but the phylogenetic signal for our measures of microhabitat use is rather weak.

Weak overall phylogenetic signal appears to result from heterogeneity between co-mimics and non-co-mimics. When considering only pairs of non-co-mimic species, distances along four of the five measured variables (FS2, FS3, topography, and flight height) were significantly positively correlated with phylogenetic distances (Figure 2), resulting in a significant phylogenetic signal in global microhabitat (*r =* 0.176, *n* = 1,206, *p* < 0.0001). By contrast, among co-mimics we failed to detect a significant positive correlation for any of the microhabitat variables (Figure 2). Mimicry thus obscures patterns of phylogenetic signal in microhabitat niche.

We carried out a series of tests to specifically investigate the hypothesis that this pattern was due to greater ecological similarity among co-mimics than expected given phylogenetic relationships. Testing this hypothesis requires controlling for phylogeny. Phylogenetic signal can either be removed from the ecological data (regression-based methods) or incorporated into the expected distribution of type I error (simulation-based methods). The first category of methods showed a significant positive correlation between mimicry distances and ecological distances for FS1, flight height, and global microhabitat when phylogeny was controlled for in partial Mantel tests (Table 1), indicating that species in the same mimicry groups tend to be ecologically more similar than species in different mimicry groups. A follow-up analysis, in which ecological distances were regressed onto phylogenetic distances and the resulting residuals tested for convergence (negative values) and divergence (positive values) among co-mimics and non-co-mimics, respectively, confirmed that this pattern of ecological similarity among co-mimics and ecological dissimilarity among non-co-mimics was stronger than expected given the phylogeny (Table 1). Finally, simulations of evolution of the microhabitat variables on the phylogeny under both gradual and speciational models of character evolution also showed that co-mimics were more similar and non-co-mimics more dissimilar than expected assuming no selection and given the phylogeny along FS1, flight height, and global microhabitat (Table 1). Thus, the observation of convergent microhabitat use among co-mimics is robust to both simulation and regression analyses, each based on a different set of assumptions.

**Table 1:**
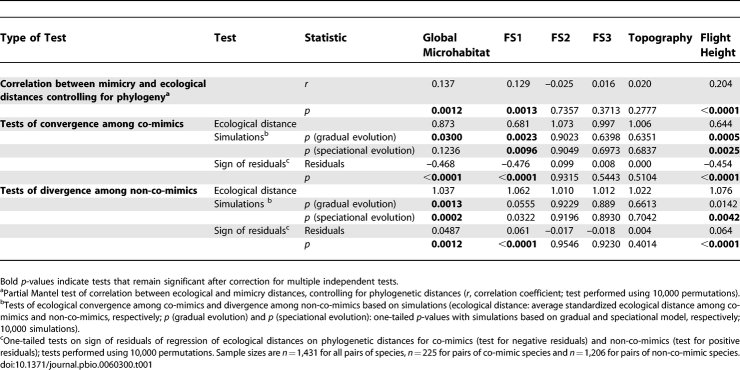
Tests of Adaptive Association Between Mimicry and Ecological Variables

Increased ecological similarity among co-mimics is likely to be due initially to mimicry evolving among spatially co-occurring species (i.e., species with at least partial microhabitat niche overlap), followed by further ecological convergence to maximize the efficiency of the warning signal. Divergence among non-co-mimics could be a byproduct of convergence among co-mimics, but could also be enhanced by competitive interactions (niche partitioning). Although the microhabitat variables measured here do not represent resources per se, they are likely correlated with key limiting resources, including adult pyrrolizidine alkaloids and food sources, lek sites or larval host-plants [24,27].

To investigate whether interspecific competition occurred along the variables measured and to determine the interplay between competition and mimicry we used niche complementarity, whereby species that are similar along one niche axis diverge along another [36], as an indicator of competition. As only co-mimics benefit from occurring together, niche complementarity is expected to be greater among non-co-mimics. In agreement with these predictions, significant negative correlations between two microhabitat variables were detected only among non-co-mimics, for FS1–FS3, FS2–FS3 and FS1–flight-height (Table 2). Corresponding correlations for co-mimics were not significantly different from zero and correlation coefficients were positive (Table 2). This provides evidence for competitive interactions between non-co-mimic species only, showing that mimicry counteracts the effects of competition along the variables measured by promoting co-occurrence. Nonetheless, although all tests performed produced similar correlation coefficients, the significance of niche complementarity among non-co-mimics was not robust to the different tests. This was most probably due to reduced power to detect effects in the simulation analysis caused by the inherent increase in complexity and stochasticity associated with this method (unlike the previous tests, here two variables were simulated simultaneously). This also suggests that niche complementarity along microhabitat variables is a weak phenomenon relative to the convergence observed between co-mimics.

**Table 2:**
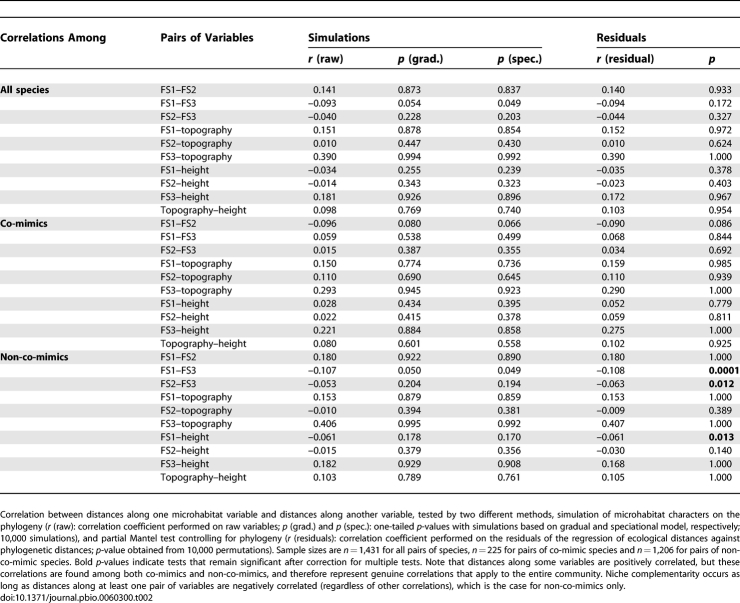
Tests for Niche Complementarity (One-Tailed Tests for Negative Correlations)

Although previous studies have shown that competitive interactions among species can alter community-level patterns of phylogenetic niche conservatism and cause overdispersion [7–9,37], we provide evidence for the opposite pattern, namely adaptive, increased ecological similarity locally between species that benefit from co-occurrence. Mutualistic and commensal interactions that could lead to similar ecological convergence are widespread among plants [38], animals [39–41], and microorganisms [11]. For example, vertebrates commonly form mixed-species groups or flocks, thereby enhancing protection against predators [40,42–44] and foraging efficiency [40]. Species involved in mixed-species groups should have similar habitat requirements [40] and are therefore a nonrandom subset of species of the community. Furthermore, such associations should prevent habitat divergence and could also promote increased habitat similarity, provided that the costs of competition are outweighed by the benefits of coexistence. Facilitative interactions among plants via shared pollinators, shared seed dispersers, or shared seed predators might also promote adaptive convergence in several ways. Plant species that are pollinated by the same pollinator guild often show dramatic convergence in flower morphology [45,46], in a manner analogous to Müllerian mimicry, because coexisting species benefit from the increased abundance of pollinators. Such species are also likely to show habitat convergence similar to that observed here, as well as increased synchrony of flowering time [47,48]. This would act to maximize overlap with pollinators, provided that the benefits outweigh the costs of increased competition and cross-pollination. In addition, synchrony in flowering time and seed set could also be promoted by limitation in seed dispersers [49] or to ensure satiation of generalist seed predators [47,49,50]. Our study demonstrates a tight link between the evolution of a mutualism and the evolution of microhabitat niche, which implies a long history of ecological interactions between members of local communities. If pervasive, such effects would imply that local ecological communities are adaptively assembled to a greater and more complex degree than is commonly considered.

Determining the factors that shape local species assemblages is a hot topic in modern community ecology [51,52]. In addition to interactions among species, local assemblages may also be determined by neutral processes, such as dispersal limitation and drift, or by habitat filtering, whereby only species adapted to a certain habitat can colonize, and each of these processes can have different relative importance. This field has recently benefited from the development of neutral theories of diversity [53–56] and the incorporation of phylogenetic information into community ecology [4]. Both approaches facilitate the detection of non-neutral processes [57]. However, despite positive interactions among species being widespread [12,14,15,40,41], the majority of theoretical and empirical studies that incorporate phylogenetic information have considered only habitat filtering and competitive interactions [4,8,10,57]. One notable exception is a recent study of Mexican woody plant communities, which demonstrated that facilitation turns to competition with increased phylogenetic relatedness [58]. This involved a study of the relationship between phylogenetic relatedness and temporal patterns of co-occurrence among species from the same community. The approach was very different to that taken here, in which species of similar levels of relatedness are either mutualistic (co-mimics) or not (non-co-mimics). More generally, community ecology theory would greatly benefit from expanding current phylogenetic models [57] to incorporate positive interactions alongside neutral processes, habitat filtering, and competition in order to derive testable predictions and disentangle these processes.

Finally, our findings further support the idea of mimicry as a driver of speciation through ecological adaptation [59]. The implications of mimicry evolution extend far beyond the evolution of warning signals, with links to microhabitat use, including flight height and forest structure [24–26], but also to mate choice [60], flight physiology [61], and larval host-plant use [24,27]. Here we show that the links between mimicry and microhabitat use are a clear result of adaptation. Our results therefore support the hypothesis that shifts in mimicry trigger shifts in microhabitat and vice versa. Divergence in both factors contributes to prezygotic reproductive isolation and ultimately speciation through assortative mating [60,62], while shifts in mimicry additionally cause postzygotic isolation through selection against hybrids with intermediate, non-mimetic wing patterns [63,64]. Hence multiple coevolved factors may act together to generate reproductive isolation.

## Materials and Methods

### Ecological data.

The field site for this study covered an area of ∼15 km^2^ on the south bank of the Napo River, Ecuador (∼0°32′S, 76°24′W) [35]. Field work was conducted between October and December 2005. Sixteen variables representing forest structure (Table S2) were measured in 18 circular plots (diameter: 30 m) established in different areas of the study site. A principal component analysis was then performed on these variables to extract meaningful information on forest structure (Table S2). The first three principal components (FS1: dense understorey, abundance of vines, 25.1%; FS2: abundance of palms, absence of trees, 18.1%; and FS3: open canopy, palms variable, 14.5%) were retained. Eight of these plots comprised paired adjacent ridge and valley plots to measure topographic influence. Ithomiines were surveyed in each study plot, captured with a hand net, identified, and either marked and released or kept for genetic analyses. Height at initial observation was recorded [65], and the individual was attributed measures of forest structure and topography (0: valley, 1: ridge) for the plot of capture. A total of 1,231 individual butterflies were surveyed and included in the analyses. Averages were calculated for each variable for each species, and distance matrices for each variable computed. Global microhabitat distances were calculated as the Euclidean distances between species in the 5-dimensional ecological space defined by the five centered and scaled microhabitat variables measured. Each species was classified among eight mimicry complexes [27,59] to generate a mimicry distance matrix (0: co-mimics, 1: non-co-mimics).

### Phylogenetic analyses.

We generated a phylogeny of the 58 ithomiine species (24 genera) of the community using Bayesian Inference under the uncorrelated lognormal relaxed clock model implemented in BEAST [66], using a 2,296 bp mitochondrial region spanning the *CoI*, *tRNA leu* and *CoII* genes, as well as a 1,215 bp fragment of *Elongation Factor 1a*, a nuclear gene (Table S1) [35]. Each region followed a GTR+Γ model of substitution, and two MCMC chains were run for 200 million generations (sampling every 1,000 generation, 10% burn-in). Two species had two subspecies each with different color patterns in the community, which were considered as separate taxa in both cases (Figure 1). To increase the accuracy of the tree topology we added published sequences of genera that were not represented in the community [67,68], but these additional taxa were pruned from the tree prior to the analyses presented below. Six rare ithomiine species were also excluded from the analyses because of missing ecological data (Figure 1).

### Statistical analyses.

All analyses on the phylogeny were performed on the maximum clade credibility tree with average branch lengths, computed by TreeAnnotator [66] from the 360,000 trees retained. To account for phylogenetic uncertainty we collapsed all nodes that had a posterior probability less than 0.90. Analyses were conducted on all 54 taxa for which sufficient ecological data was recorded. All statistical analyses were performed with R [69], using packages APE 2.1–3 [70], Geiger 1.2–06 [71], Vegan 1.11–0 [72], and Cluster 1.11.10 [73].

To investigate ecological segregation of mimicry complexes we performed a MANOVA on all variables and an ANOVA for each individual variable. These analyses were performed on the entire data set to test whether mimicry complexes are ecologically segregated (regardless of species identity), and on species' averages to test whether co-mimetic species are more similar than species picked at random (regardless of species abundance). The significance of ANOVA/MANOVA test statistics was assessed by permuting the observed ecological data (10,000 permutations).

Correlations between phylogenetic and ecological distances among all species, among co-mimics and among non-co-mimics were computed and their significance was tested by permuting the observed ecological data among species (10,000 permutations, one-tailed test for positive correlation). We performed a partial Mantel test for the correlation between ecological and mimicry distances while controlling for phylogeny. We did this by first regressing ecological and mimicry distances against phylogenetic distances and then assessing the significance of correlations between residual ecological and mimicry distances by permutation of the residual distances among species pairs (logistic regression for mimicry distances; 10,000 permutations, one-tailed test).

Tests for ecological convergence and divergence for each microhabitat variable and for the global microhabitat were performed in two additional ways: (1) simulated character evolution, which incorporates phylogeny into the expected distribution of type I error, and (2) regression-based methods, which remove the phylogenetic component of variation in ecological characters. In the first way, we calculated the average ecological distance among co-mimics standardized by the total tree ecological distance (tests for ecological convergence among co-mimics), or the average distance among all non-co-mimics standardized by the total tree ecological distance (tests for ecological divergence between mimicry complexes). Character evolution was then simulated on the phylogeny assuming gradual and speciational character evolution (10,000 simulations), and the actual value of the parameters of interest compared with the distribution of the same parameter generated by the simulations to obtain *p*-values. As we wanted to test whether scaled ecological distances were smaller than expected among co-mimics and greater than expected among non-co-mimics, one-tailed tests were used in each case. In the second way, ecological distances were regressed against phylogenetic distances using all species. We then permuted these residual ecological distances among species pairs to determine whether the observed residuals were more negative or positive then expected by chance for co-mimics and non-co-mimics, respectively (10,000 permutations).

Niche complementarity was investigated by calculating the correlation coefficient of distances along one ecological variable with distances along another variable, for all pairs of variables. The significance of correlation coefficients was tested in two ways. First, as above, the actual value was compared with the distribution of the same parameter generated from 10,000 replicate simulations of character evolution under both gradual and speciational models. Second, a partial Mantel test was carried out for each pair of ecological variables, in which distances along each of the two variables were regressed against phylogenetic distances. We assessed the significance of correlations between residuals for each pair of ecological variables by permutation of the residual distances among species pairs. The tests were one-tailed (negative correlations) and the significance of each test was based on 10,000 permutations.

We controlled for multiple tests using the false discovery rate procedure [74], a powerful alternative to the Bonferroni correction that seeks to minimize both type I and type II errors, with the allowed proportion of false positives set at 0.05. Unless stated otherwise, all tests presented in the text, tables and figures are significant after this correction.

## Supporting Information

Table S1 List of Specimens Used, with GenBank Accession Numbers (36 KB PDF)

Table S2 Principal Component Analysis of Forest Structure Variables (28 KB PDF)
