# Supplementary material for: Mutualistic Interactions Drive Ecological Niche Convergence in a Diverse Butterfly Community
Source: PLoS Biol. 2008 Dec 2;6(12):e300. doi: 10.1371/journal.pbio.0060300 (PMC2592358; doi:10.1371/journal.pbio.0060300)
Supplement: Supplementary file 1 [file pbio.0060300.st001.pdf]

List of specimens used with GenBank accessions.

**Local species**[1]	**mimicry**	***CoI-CoII***	***EF1α***
Aeria eurimedia	eurimedia	EU069022	EU069114
Brevioleria arzalia	aureliana	EU068977	EU069119
Brevioleria seba	aureliana	EU069025	EU069120
Callithomia alexirrhoe	hermias	EU069027	EU069122
Callithomia lenea	confusa	EU069032	EU069126
Ceratinia tutia	hermias	EU068979	EU069131
Dircenna dero	confusa	EU069034	EU069133
Episcada sulphurea	eurimedia	EU069036	EU069135
Forbestra equicola	hermias	EU068980	EU069137
Forbestra olivencia	hermias	EU068981	EU069141
Forbestra proceris	hermias	EU069041	EU069144
Godyris zavaleta	eurimedia	EU068983	EU069147
Heterosais nephele	agnosia	EU068984	EU069149
Hyalyris spnov	hermias	EU069044	EU069150
Hypoleria lavinia	aureliana	EU068985	EU069152
Hyposcada anchiala	hermias	EU068986	EU069154
Hyposcada illinissa	lerida	EU068963	EU069156
Hyposcada kena	lerida	EU069051	EU069161
Hypothyris anastasia	hermias	EU068987	EU069165
Hypothyris euclea	hermias	EU068988	EU069167
Hypothyris fluonia	hermias	EU069021	EU069169
Hypothyris mamercus	mamercus	EU068989	EU069171
Hypothyris moebiusi	hermias	EU069062	EU069176
Hypothyris semifulva	hermias	EU068965	EU069178
Ithomia agnosia	agnosia	EU069066	EU069181
Ithomia amarilla	eurimedia	EU068992	EU069185
Ithomia salapia	eurimedia	EU069069	EU069187
Mechanitis lysimnia	hermias	EU068993	EU069189
Mechanitis mazaeus	hermias	EU068996	EU069196
Mechanitis messenoides	mamercus	EU068995	EU069194
Mechanitis messenoides	mothone	EU068995	EU069194
Mechanitis polymnia	hermias	EU068966	EU069199
Melinaea marsaeus	mamercus	EU069077	EU069201
Melinaea marsaeus	mothone	EU069077	EU069201
Melinaea menophilus	mamercus	EU069001	EU069203
Melinaea satevis	hermias	EU069003	EU069206
Methona confusa	confusa	EU069080	EU069208
Methona curvifascia	confusa	EU068974	EU069210
Methona grandior	confusa	EU068957	EU069214
Napeogenes achaea	mamercus	EU069084	EU069216
Napeogenes duessa	mamercus	EU069006	EU069219
Napeogenes aethra	hermias	EU069008	EU069223
Napeogenes inachia	eurimedia	EU069007	EU069221
Napeogenes pharo	eurimedia	EU069009	EU069226
Napeogenes quadrilis	hermias	EU069010	EU069228
Napeogenes sylphis	aureliana	EU069011	EU069230
Oleria agarista	lerida	EU069093	EU069232
Oleria assimilis	lerida	EU069012	EU069235
Oleria gunilla	lerida	EU069097	EU069238
Oleria ilerdina	lerida	EU069099	EU069240
Oleria onega	lerida	EU068970	EU069241
Oleria sexmaculata	lerida	EU069100	EU069244
Pseudoscada florula	aureliana	EU069101	EU069247
Pseudoscada timna	agnosia	EU069015	EU069249
Pteronymia primula	eurimedia	EU069016	EU069254
Pteronymia sao	eurimedia	EU069105	EU069255
Pteronymia vestilla	eurimedia	EU069017	EU069259
Scada zibia	eurimedia	EU069018	EU069261
Thyridia psidii	confusa	EU069019	EU069263
Tithorea harmonia	hermias	EU069020	EU069265

**Additional species**[1-3]	***CoI-CoII***	***EF1α***
Athesis clearista	DQ157476	DQ177923
Athyrtis mechanitis	DQ069230	DQ073022
Elzunia pavonii	DQ069234	DQ073026
Episcada hymenaea	DQ157491	DQ177937
Greta andromica	DQ157496	DQ177942
Greta polissena	DQ157498	DQ177944
Hyalenna paradoxa	DQ157486	DQ177932
Megoleria susiana	DQ157516	DQ177962
Oleria aegineta	DQ157526	DQ177972
Oleria rubescens	DQ085460	DQ085455
Pagyris ulla	DQ157530	DQ177975
Paititia neglecta	DQ073038	DQ073034
Patricia dercyllidas	DQ157531	DQ177976
Placidina euryanassa	DQ157532	DQ177977
Pteronymia tucuna	DQ157542	DQ177987
Pteronymia veia	DQ069242	DQ073036
Sais rosalia	DQ157543	DQ177988
Veladyris pardalis	DQ157547	DQ177993
Velamysta phengites	DQ157548	DQ177994
Tellervo zoilus (Tellervinae)	DQ157549	DQ177995

[1] Elias et al. (2007) *Proc. Roy. Soc. B* **274**, 2881-2889
[2] Brower et al. (2006) *Syst. Entom.* **31**, 288-301
[3] Whinnett et al. (2005) *Ann. Entomol. Soc. Am.* **98**, 873-886
