# Supplementary material for: Mutualistic Interactions Drive Ecological Niche Convergence in a Diverse Butterfly Community
Source: PLoS Biol. 2008 Dec 2;6(12):e300. doi: 10.1371/journal.pbio.0060300 (PMC2592358; doi:10.1371/journal.pbio.0060300)
Supplement: Supplementary file 2 [file pbio.0060300.st002.pdf]

Principal Component Analyses on forest structure variables

We measured 8 variables in each plot (canopy cover, ground cover, numbers of palms, vine and trees, tree diameter, and two measures of understorey vegetation density). Each variable was measured in 4 sectors of the plot, such that the average (_m) and the standard deviation (_sd) could be computed for each plot, the latter indicating variability within the plot.

Variable loading

	PC1 = FS1	PC2 = FS2	PC3 = FS3	PC4	PC5	PC6	PC7	PC8	PC9	PC10	PC11	PC12	PC13	PC14	PC15	PC16
**canopy_cover_m**	-0.229	-0.034	-0.644	0.135	0.052	0.601	0.074	0.363	0.024	0.035	-0.014	0.067	-0.034	0.066	-0.021	0.005
**ground_cover_m**	0.33	-0.149	0.504	0.212	-0.647	0.143	-0.143	0.026	0.176	0.148	0.23	0.017	0.046	0.029	-0.028	-0.004
**palms_m**	0.207	0.726	-0.032	0.451	-0.155	0.145	-0.098	-0.297	-0.231	0.12	0.036	0.065	-0.066	0.059	0.034	0.006
**vine_m**	0.79	-0.225	-0.422	-0.14	0.159	-0.03	0.067	-0.188	0.021	-0.017	0.04	-0.201	-0.058	0.119	-0.01	-0.01
**tree_m**	-0.558	-0.607	-0.027	0.259	-0.029	-0.085	0.388	0.07	-0.032	-0.075	0.258	-0.107	0.046	0.028	0.043	0.004
**tree_diam_m**	0.214	0.707	-0.224	0.225	-0.015	-0.331	0.086	0.35	-0.211	0.168	0.072	-0.185	0.02	-0.044	-0.018	-0.002
**stickmark_m**	-0.705	0.37	-0.155	-0.313	-0.154	-0.237	0.157	0.094	0.198	0.266	-0.06	0.106	-0.02	0.061	0.029	-0.009
**vegdist_m**	-0.707	0.077	0.354	-0.375	0.25	0.007	0.22	-0.23	-0.066	0.201	0.107	-0.054	-0.058	0.018	-0.044	0.009
**canopy_cover_sd**	0.64	-0.501	0.086	-0.138	-0.073	-0.359	0.176	0.106	-0.269	0.064	-0.079	0.205	0.081	0.074	-0.012	0.005
**ground_cover_sd**	0.646	-0.343	0.483	0.204	0.141	-0.106	0.067	0.192	0.215	0.161	-0.112	-0.057	-0.171	-0.008	0.018	0.008
**palms_sd**	0.194	0.616	0.539	-0.071	-0.099	0.226	0.352	0.019	0.106	-0.106	-0.2	-0.134	0.137	0.052	0.005	0.004
**vine_sd**	0.702	-0.065	-0.453	-0.229	0.25	0.18	-0.003	-0.129	0.146	0.285	0.08	0.002	0.144	-0.061	0.02	0.005
**tree_sd**	-0.151	-0.2	0.326	0.677	0.521	0.145	0.204	-0.079	-0.03	0.138	-0.061	0.078	0.053	-0.009	-0.012	-0.012
**tree_diam_sd**	0.422	0.545	-0.311	0.196	0.06	-0.249	0.404	-0.093	0.257	-0.193	0.135	0.167	-0.042	-0.021	-0.02	0.002
**stickmark_sd**	0.526	0.142	0.374	-0.479	-0.003	0.39	0.305	0.114	-0.206	-0.031	0.134	0.075	-0.083	-0.051	0.018	-0.008
**vegdist_sd**	0.068	0.415	0.44	-0.127	0.592	-0.085	-0.39	0.207	0.067	-0.091	0.196	0.055	0.044	0.073	0.01	0.001
**% of Variance**	**25.136**	**18.084**	**14.506**	**9.396**	**8.001**	**6.514**	**5.5**	**3.631**	**2.745**	**2.316**	**1.787**	**1.345**	**0.663**	**0.314**	**0.058**	**0.004**
**Cumulative %**	**25.136**	**43.22**	**57.726**	**67.122**	**75.123**	**81.637**	**87.137**	**90.768**	**93.513**	**95.829**	**97.616**	**98.962**	**99.624**	**99.938**	**99.996**	**100**

We retained the three first components for the analyses (named FS1, FS2 and FS3 throuhgout the text):

FS1: dense understorey, abundance of vines

FS2: abundance of palms, absence of trees

FS3: open canopy, palms variable
